# Mechanism of action of gut microbiota and probiotic *Lactobacillus rhamnosus* GG on skeletal remodeling in mice

**DOI:** 10.1002/edm2.440

**Published:** 2023-07-28

**Authors:** Abdul Malik Tyagi

**Affiliations:** ^1^ Division of Endocrinology CSIR‐CDRI Lucknow India

**Keywords:** bone mass, gut microbiota, *Lactobacillus*, osteoporosis, sex steroid

## Abstract

**Introduction:**

Gut microbiota (GM) is the collection of small organisms such as bacteria, fungi, bacteriophages and protozoans living in the intestine in symbiotics relation within their host. GM regulates host metabolism by various mechanisms.

**Methods:**

This review aims to consolidate current information for physicians on the effect of GM on bone health. For this, an online search of the literature was conducted using the keywords gut microbiota, bone mass, osteoporosis, *Lactobacillus* and sex steroid.

**Results and Conclusions:**

There is a considerable degree of variation in bone mineral density (BMD) within populations, and it is estimated that a significant component of BMD variability is due to genetics. However, the remaining causes of bone mass variance within populations remain largely unknown. A well‐recognized cause of phenotypic variation in bone mass is the composition of the microbiome. Studies have shown that germ‐free (GF) mice have higher bone mass compared to conventionally raised (CR) mice. Furthermore, GM dysbiosis, also called dysbacteriosis, is defined as any alteration in the composition of the microbial community that has been colonized in the host intestine and associated with the development of bone diseases. For instance, postmenopausal osteoporosis (PMO) and diabetes. GM can be modulated by several factors such as genetics, age, drugs, food habits and probiotics. Probiotics are defined as viable bacteria that confer health benefits by modulating GM when administered in adequate quantity. *Lactobacillus rhamnosus* GG (LGG) is a great example of such a probiotic. LGG has been shown to regulate bone mass in healthy mice as well as ovariectomized (OVX) mice via two different mechanisms. This review will focus on the literature regarding the mechanism by which GM and probiotic LGG regulate bone mass in healthy mice as well as in OVX mice, a model of PMO.

## Graphical abstract

Gut microbiota (GM) is the collection of small organisms such as bacteria, fungi, bacteriophages and protozoans living in the intestine in symbiotics relation within their host. GM regulates host metabolism through various mechanisms. The skeletal system is one of the systems regulated by GM. This review is focussing the mechanism by which GM regulates bone mass.

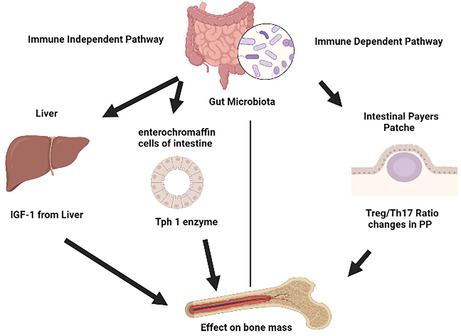

## 1 INTRODUCTION

Metabolic syndrome (MS) means the conjunction of numerous known cardiovascular risk factors, including insulin resistance, obesity, atherogenic dyslipidemia and hypertension. These conditions are interrelated and share underlying mediators, mechanisms and pathways.^1^ Analysis of this complex dynamic system using simulation and model‐based approach can facilitate the delineation of the causal pathways that lead to the emergence of the metabolic syndrome simulation and modeling of MS has been reviewed in detail in the referenced articles.^2^, ^3^, ^4^ All prokaryotic animal forms live in a symbiotic relationship with a diverse microbial community that is mainly composed of bacteria but also has Fungi, viruses, archaea and protozoa.^5^ These microorganisms usually cover all exposed surfaces of the body for instance skin and mucosal surfaces. However, a significant amount of microbiome resides along the alimentary canal from the mouth cavity to the anal opening. Traditionally, Gut microbiota (GM) was considered to affect the physiology and pathophysiology of the digestive system and immune systems. However, recently a plethora of studies showed that GM affects the metabolism and pathophysiology of multiple organs system. Thus, the traditional human‐centered view of the GM as pathogens and only immunological threats has been changed by an assessment of its primarily beneficial impact on human health.

The ‘healthy’ GM is dominated by anaerobic bacteria, with an increase of 100–1000 times that of aerobic and free‐functional anaerobic bacteria. In total, the intestinal microbiota is composed of about 500–1000 species, which are interestingly located only in a small number of known bacterial phyla.^6^, ^7^ The most abundant phyla in the human intestine are Bacteroidetes and Firmicutes, the remaining species present are the members of phyla Proteobacteria, Verrumicrobia, Actinobacteria, Fusobacteria and Cyanobacteria.^6^, ^7^ Two patterns of microbial distribution can be found throughout the gastrointestinal tract. First, microbial density increases both from the proximal to the distal gut (the stomach contains 10 microbial cells/g of content, the duodenum 1000 cells/g, the jejunum 10,000 cells/g, the ileum 10^7^ cells/g and the colon up to 10^12^ cells/g).^8^ Second, bacterial diversity surges in a similar magnitude and manner as microbial density.^8^ Many bacterial species are present in the lumen, whereas fewer, but well‐adapted species, including several proteobacteria and Akkermansia muciniphila, adhere and reside within the mucus layer close to the tissue and these are called mucosal microbiota.^9^, ^10^ Colonisation of the host begins during birth and the composition of the microbiota changes throughout host development.

Maternal transmission is the key determinant of the composition of the GM in newborns.^11^ The type of delivery of newborn babies also impacts the colonisation of the microbiome as babies born via vaginal delivery are colonized with healthy microbiome whereas babies born with cesarean section are colonized with the disrupted transmission of maternal Bacteroides strains, and high‐level colonisation by opportunistic pathogens associated with the hospital environment including Enterococcus, Enterobacter and Klebsiella species.^12^ In children, the GM reaches an adult‐like composition by ~3 years of age, through maternal transmission and close cohabitation.^11^ This phenomenon is known as microbial inheritance.^13^ By age ~3, the GM becomes resistant to colonisation by new organisms.^14^ Indeed, once acquired, the majority of strains are retained in an individual for decades.^14^ Thus, early gut colonizers, once established, have the potential to exert their biological effects on the host's health for most and perhaps all of the host's adult life.^11^ The effect of a strain's residency may take decades to manifest itself.^13^ Long‐lasting modifications of the microbiota require permanent, significant dietary changes, major changes in the host's health status or extensive manipulations, such as long‐term antibiotic treatment.^15^ Microbial inheritance occurs preferentially between family members and much less between unrelated individuals.^13^ Attesting to the relevance of microbiota inheritance, the relative risk of Crohn's disease,^16^ rheumatoid arthritis^17^ or multiple sclerosis^18^ is highly increased when a sibling is affected, even after accounting for the genetic predisposition. By contrast, cohabitation is not sufficient to modify the gut microbiome in older individuals. This explains why transmission of diseases linked to the microbiome is unusual between spouses. Microbial inheritance is now recognized as a pivotal variable in mouse breeding as well.^19^ A well‐controlled quantitative trait loci mapping study in mice indicated that microbiota composition is affected by host genetic factors, including those involved in immune responses, as well as by environmental exposures.^20^, ^21^

In an adult intestine, a total of about 10^14^ bacterial cells are present, which is roughly 10 times the number of human cells in the body.^22^ Their combined genomes known as the microbiome contain more than 5 million genes, thus outnumbering the host's genetic potential by two orders of magnitude.^23^ This large collection of gene products delivers a varied variety of biochemical and metabolic activities to counterpart host physiology. The metabolic capacity of the GM equals that of the liver, and the intestinal microbiota can therefore be considered as an additional organ.^24^ These bacteria are essential for several aspects of host biology. For example, they enable the metabolism of otherwise indigestible polysaccharides and produce essential vitamins; they are required for the development and differentiation of the host's intestinal epithelium and immune system; they confer protection against invasion by opportunistic pathogens^25^ and they have a key role in maintaining tissue homeostasis. Recent studies have also revealed that the human microbiota influences the development and homeostasis of other host tissues, including the skeletal tissue and in achieving peak bone mass.^26^

Any perturbation in the composition of the microbiota of the host is defined as dysbiosis. Dysbiosis of GM can be healed by proper intake of prebiotics and probiotics. Probiotics are live bacteria and yeasts that are good for health, especially for the digestive system and beyond. Against the usual belief, these are not the germs that cause diseases only. Probiotics are often called ‘good’ or ‘helpful’ bacteria because they help keep your gut healthy. You can find probiotics in supplements and some foods, like yogurt. Doctors often suggest they help with digestive problems. In this review, we will discuss the mechanism by which GM and probiotic *Lactobacillus rhamnosus* GG (LGG) regulate bone mass in healthy mice as well as in ovariectomized (OVX) mice.

## 2 PHYSIOLOGICAL NEXUS BETWEEN GUT IMMUNE AND SKELETAL SYSTEM

The mature human skeleton contains 206 bones, excluding the sesamoids.^27^ Bone is a metabolically active connective tissue that provides structural support as well as simplification of movement by providing levers for muscles, protection of vital organs, pools for minerals and growth factors, regulation of mineral and acid–base homeostasis and a site for hematopoiesis.^28^, ^29^ Bone consists of matrix and cells of heterogeneous origin but restricted function concerning matrix formation, mineralisation and resorption. The local, mesenchymal origin of the cells which form the skeleton called osteoblasts contrasts with their extra skeletal, hemopoietic relatives under which bone resorption takes place are called osteoclasts. However, the functions of these two diverse populations are remarkably related and interdependent. Bone cell regulation is a complicated cascade involving a plethora of local and systemic factors, including some components of the skeletal matrices, hormones and growth factors from other organ systems including the immune system.^30^

Bone and immune systems are functionally integrated systems and together referred to as osteoimmune systems by their common niche, leading to permanent pertinent interaction between them at various anatomical and vascular contacts.^31^ This novel interdisciplinary area had convinced the scientific community that there exists a strong mutual interaction between the bone and immune systems. This complex association between the two systems has fascinated scientists since the early 1970s and laid the foundation of osteoimmunology.^32^ Through the secretion of various factors, immune cells regulate bone remodeling and play an important role in bone homeostasis.^33^ Therefore, investigators presently are more interested in the various types of immune–bone cell interactions responsible for enhanced osteoclastogenesis or osteoblastogenesis observed in bone pathologies. The connection between both the immune and skeletal systems can only be appreciated by exploring the very basics of bone physiology and pathophysiology in various diseases for instance osteoporosis, Rheumatoid arthritis (RA) and osteoarthritis;^34^, ^35^, ^36^ this interaction is not a closed system and is also highly influenced by the GM. In the last decade, several reports showed that GM affects skeletal homeostasis. This regulatory effect of GM on bone mass can be immune system‐dependent or ‐independent.

One instance of the immune‐independent action of GM is the regulation of serotonin synthesis and secretion. Serotonin (5‐hydroxytryptamine or 5‐HT) is a neurotransmitter and a hormone, most of the circulating serotonin is secreted in the gut by the enterochromaffin cells.^37^ Several reports showed that bone cells express functional serotonin receptors, and gut‐derived serotonin has been demonstrated to have negative effects on bone formation in mice.^38^, ^39^ The rate‐limiting step in serotonin synthesis in the gut is catalysed by the enzyme tryptophan hydroxylase‐1 (Tph1).^40^, ^41^ GM regulates Tph1 synthesis and secretion from intestinal cells.^42^, ^43^ The action of serotonin is limited by reuptake into epithelial cells of the intestinal mucosa and serotonergic neurons, where it is broken down.^44^ This uptake is mediated by the serotonin transporter SERT.^45^ Recent studies have reported conflicting data on the effects of serotonin on bone homeostasis by treating the mice with Tph1 inhibitor to decrease gut‐derived serotonin, studies have demonstrated that treatment with Tph1 inhibitor protected OVX‐induced bone loss; however, one study showed no effect on bone after similar treatment.^46^, ^47^, ^48^ Another example of the effect of GM is the regulation of liver IGF‐1.^49^ The report has shown that GM promoted bone formation in mice by inducing the expression of IGF‐1.^50^

**FIGURE 1:**
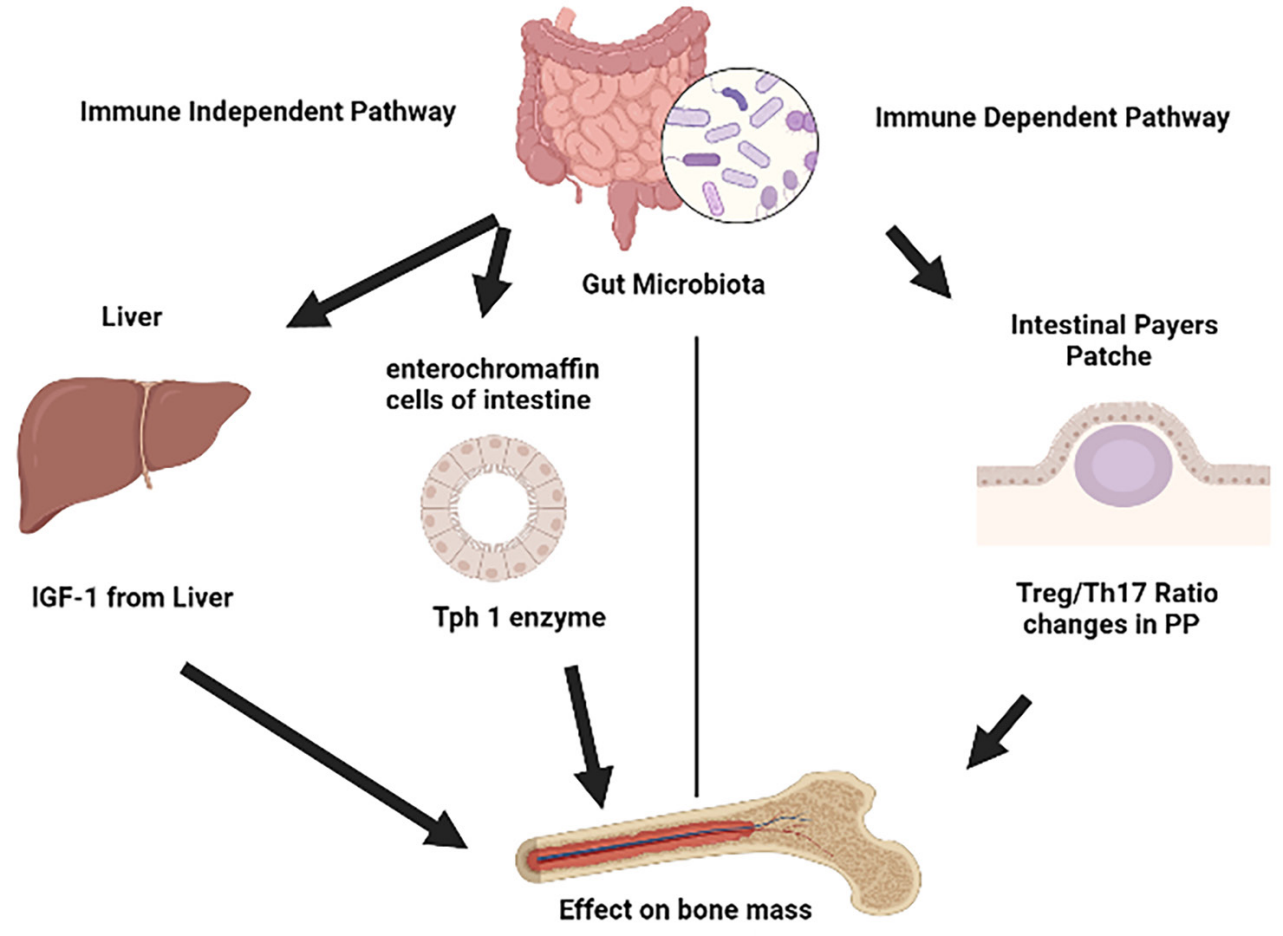
Mechanism of action of gut microbiota (GM) on skeletal homeostasis. Immune‐independent mechanism of bone mass regulation comprises of liver IGF‐1 and intestinal Tph1 synthesis and secretion. Immune‐dependent mechanism of bone mass regulation by GM is mediated by the maintenance of Treg/Th17 cells ratio, TNF‐α, and RANKL expression in the bone marrow.

The complex association of GM with the host's immune system and gut‐derived and gut‐independent growth factors and bone led investigators to investigate the impact of GM on bone mass. The effect of GM on bone has been illustrated in Figure 1. GM also can regulate skeletal homeostasis via modulating the immune system function. There are several reports produced by the scientific community showing the role of the immune system in bone mass regulation in several autoimmune, inflammatory and diabetic disorders.^51^, ^52^, ^53^ Therefore, to investigate the effect of GM on skeletal homeostasis study was conducted using GF mice because GF mice lack all microorganisms (as determined within the limitations of the detection methods available) and are housed in tightly controlled and monitored isolators to prevent contamination.

## 3 MECHANISM OF SKELETAL HOMEOSTASIS IN CONV‐R AND GF MICE

Gut‐microbiota regulates bone mass through two mechanisms primarily via immune system‐dependent and secondarily by immune system‐independent pathways.^26^, ^49^ This review will discuss both mechanisms. Klara et al. in 2012 reported that 7 weeks old female C57BL/6 GF mice had higher proximal tibial trabecular Volumetric bone mineral density (vBMD) compared to the CONV‐R mice as assessed by peripheral quantitative computed tomography (pQCT). They further examined the femur bone by microcomputed tomography (μCT). The author found that trabecular bone volume/tissue volume (BV/TV) was increased in the distal femur of GF mice compared with CONV‐R mice.^26^ The increased BV/TV was associated with an increased trabecular number (Tb. N) and decreased trabecular separation (Tb. Sp), whereas trabecular thickness (Tb. Th) was unchanged in GF compared with CONV‐R mice. Unchanged Tb. The increased Tb. N was an indicator of new bone formation in GF mice. The author further confirmed these results by dynamic histomorphometry assay as well and reported that mineralising surface per trabecular bone surface was increased in GF mice compared with CONV‐R mice. However, there was no change in the mineral apposition rate.

The positive effects of GM were not restricted to trabecular bones only as the analysis of cortical bone in the mid‐diaphyseal region of the femur by μCT showed that cortical bone area was increased in GF mice compared with CONV‐R mice. To investigate the mechanism author measured the levels of serotonin, sex steroid, and parathyroid hormone (PTH) and found the levels of sex steroids and PTH remained unchanged in GF mice.^26^ However, the author reported increased levels of serotonin in GF mice compared to CONV‐R mice. This report further analyzed the bone marrow cell population and found the numbers of BM CD4^+^ cells and osteoclast progenitor CD11b^+^ cells were decreased in GF mice. This report concludes that GF mice have more bone mass compared to CONV‐R mice due to the reduced number of CD4^+^ and osteoclast precursor CD11b^+^ cells.^26^ Surprisingly, the effect of GM on bone mass was strain‐dependent as male BALB/C mice had the opposite effect of GM on bone mass.^54^ Martin et al. studied the effect of GM on juvenile growth in male BALB/C mice and reported that the growth parameters of CONV‐R and GF infant male mice fed a standard breeding diet until 8 weeks of age. After weaning, the GF and CONV‐R animals were fed similar amounts of food relative to body weight, and yet at 8 weeks of age, GF mice weighed 14.5% less and were 4% shorter than CONV‐R mice. These growth differences were most pronounced after weaning.

Thus, with a standard breeding diet, the GM ensures optimal weight gain and longitudinal growth, especially around weaning. Remarkably, the 17% weight gain seen in CONV‐R animals was not a consequence of increased adiposity. The epididymal fat pads and adipocyte size of CONV‐R and GF male mice remained the same. Likewise, levels of leptin, a circulating marker of fat stores^55^ were similar in the sera of CONV‐R and GF animals. However, the weight gain of the organs of CONV‐R animals was greater than that of GF mice confirming that microbiota in CONV‐R mice is associated with optimal systemic somatic growth. Interestingly, these results were opposite to the increased adiposity that results from subtherapeutic antibiotic treatment in infant mice that is caused by disrupting the GM community.^56^, ^57^ CONV‐R mice were 4% longer, indicating that the microbiota also influences skeletal growth. Bone growth parameters, including femur length, cortical thickness, cortical bone fraction, and the trabecular fraction of the femur were all reduced in GF mice, although cortical bone mineral density (BMD) remained unaffected between the groups.

The overall author showed that the GM sustains postnatal somatic tissue growth, leading to increased bone mass gain and enhanced longitudinal growth. In another study, Jing Yan et al. studied the effect of GM on bone mass in 2‐month‐old sex‐matched GF F1 hybrid CB6F1 stain generated from female BALB/C and male C57BL/6 mice. They reported that colonisation of GF mice with a specific pathogen‐free (SPF) microbiome induces both bone resorption and formation, with the overall outcome of colonisation fluctuating with the period of colonisation. Although colonisation of adult mice acutely reduces bone mass, the colonisation of 2 months old GF mice with SPF microbiome for 1 month induces bone loss in femur trabecular bone as assessed by μCT. Serum levels of C‐terminal telopeptides of type‐I collagen (CTX) (a marker of bone resorption) and procollagen type I N‐terminal propeptide (P1NP) (a marker of bone formation) were higher in colonized mice compared to GF mice. However, when 2 months old GF mice were colonized with SPF microbiome and analysed after 8 months post‐colonisation. An increase in bone formation and growth plate activity was observed, resulting in increased longitudinal and radial bone growth.^49^

Interestingly, femur length was significantly longer in colonized mice of both sexes compared with littermate GF mice. This effect was also observed in the spine. The height of L5 vertebrae was also greater in colonized mice. Long‐term colonisation also increased the periosteal and endosteal area compared with GF siblings, without changing cortical thickness or porosity. although the effect on radial growth was less pronounced in females. This data suggested that colonisation promotes radial and longitudinal bone growth, consistent with the more active endochondral ossification seen after short‐term colonisation. The observed increase in bone size after long‐term colonisation is accompanied by a modest increase in body weight. The author, also found that in contrast to short‐term colonisation, long‐term colonized mice had a trend toward increased trabecular bone mass in the femur and have comparable bone mass in L5 vertebrae. This suggests that during long periods of colonisation, the bone formation‐promoting effects of colonisation overtake the effect on bone resorption. Consistent with this, increased bone resorption in response to colonisation appears to be transient, as serum CTX in long‐term colonized and GF mice were the same. Eight months after colonisation, bone formation markers either by serum P1NP or by dynamic histomorphometry were low, and the author was unable to detect significant differences in long‐term colonized mice compared with GF littermates.^49^

Mechanistically, the author found the levels of IGF‐1 were higher in long‐term colonized mice as compared to GF mice. IGF‐1 is a potent inducer of bone formation.^58^, ^59^, ^60^, ^61^, ^62^ The levels of IGF‐1 were reported higher in CONV‐R mice compared to GF mice.^54^ The author reported that the levels of IGF‐1 were higher in both short‐term as well as long‐term colonized mice compared to GF mice. The bioactivity of IGF‐1 is modulated by IGF‐binding proteins (IGFBPs) that have a higher affinity for IGF‐1 than the IGF‐1 receptor, and inhibit the biological effects of IGF‐1 through sequestration.^59^ The majority of serum IGF‐1 is bound to IGFBP3.^63^ No difference in the level of serum IGFBP3 at either point after colonisation. Therefore, colonisation likely increases free IGF‐1. Consistent with this, the expression of runt‐related transcription factor 2 (Runx2), a downstream target of the IGF‐1 signaling pathway in bone, was increased in epiphyseal bone samples from colonized mice. As circulating IGF‐1 is regulated by growth hormone (GH),^64^ Therefore, the author examined GH levels in colonized mice but found no difference in the levels of GH between colonized and GF mice. As shown by the data the possible mechanism by which microbiota could promote bone formation in adult mice is by increasing serum levels of IGF‐1.

Colonisation of adult mice was sufficient to increase serum IGF‐1 compared with GF siblings. As many factors, including age and nutrition,^65^, ^66^ affect IGF‐1 levels, sibling controls fed by identical diets demonstrate that microbiota colonisation increases serum IGF‐1. This is further supported by antibiotic treatment studies, in which sterilisation of the gut decreased IGF‐1. Although circulating IGF‐1 is made primarily by the liver, IGF‐1 synthesis is ubiquitous. Colonisation increased both liver and adipose tissue IGF‐1. In Drosophila, colonisation enhanced the activity of Drosophila insulin/IGF‐like peptide in the fat body, a liver‐ and adipose‐like organ,^67^ suggesting that the pathways by which microbiota influence IGF‐1 production are likely highly conserved. Paracrine/autocrine actions of locally produced IGF‐1 may be also modulated by colonisation, as the mRNA expression of the Igf1 gene was increased in the bone marrow. Therefore, these data strengthened the link between GM and IGF‐1 and bone formation by demonstrating that manipulation of the microbiota in adult mice alters serum IGF‐1 levels, with durable effects observed after long‐term colonisation. The mechanism of GM on bone has been illustrated in Figure 1.

Biology is a science of exception and this is true for osteomicrobiology as well. Therefore, it is very important to mention here that Darin at all reported that the reconstitution of GM in GF mice did not cause bone loss in mice. They studied the impact of GM colonization on bone mass in an outbred strain of mice (Swiss Webster) and an inbred strain (C57BL/6) of mice. GF mice displayed a high degree of colonization, as indicated by more than 90% of the operational taxonomic units present in the starting inoculum being successfully colonized in the mice when they were examined at the end of the experiment. Despite the successful colonization of GF mice with the GM of either mouse or human origin, the bone mass did not change significantly in any of the groups tested. Furthermore, static and dynamic bone parameters and osteoclast precursor and T‐cell populations, as well as the expression of several inflammatory markers, were mostly unchanged following microbial colonization of GF mice.^68^ Discrepancies in the results from this study with other published studies may be attributed due to these factors. First, the mode of transplantation of the microbiota differed. Darin at al conventionalized their mice with caecal contents. However, as reported by Sjögren et al.,^26^ their conventionalization process was by coprophagy, where caecal contents were put onto the fur of the mice, whereas Darin's group conventionalized their mice by intragastric gavage after the caecal contents were prepared and maintained under anaerobic conditions. While it is hard to speculate on whether this ultimately culminated in pronounced differences in community colonization, it cannot be excluded. Another possible explanation for the observed differences is the inherent differences between the microbiotas used to conventionalize mice manifesting in different bone responses. It has been well documented that mice purchased from different animal vendors and housed at different mouse facilities foster microbial community colonization that is location specific.^69^

## 4 MECHANISM OF BONE MASS REGULATION VIA DIFFERENTIAL GM IN CONV‐R MICE

There is considerable natural variation in peak BMD within populations, with most of the variation being attributed to genetic heterogeneity.^70^, ^71^, ^72^ However, the remaining nongenomic factors that contribute to peak BMD variance remained uncertain. A well‐recognized determinant of phenotypic variability within populations is the composition and community structure of the gut microbiome.^73^, ^74^ Additional evidence showing the regulation of bone mass by GM in healthy mice comes from Taconic (TAC) mice and Jackson (JAX) mice. Despite having the same genetic background age‐matched male or female C57BL/6 mice from TAC had lower bone mass as compared to the JAX mice.^75^ We have reported that JAX mice have higher femoral BV/TV, Tb. Th, Tb. N and lower Tb. Sp compared to the TAC mice. We reported that TAC mice harbor a relatively more inflammatory microbiome compared to JAX mice.

The high inflammatory nature of the GM colonized in TAC mice was attributed due to the presence of segmented filamentous bacteria (SFB), which are spore‐forming, Gram‐positive commensal bacteria. In the mouse, SFB drives the development and expansion of Th17 cells that are produced and reside in the intestinal lamina propria.^76^, ^77^, ^78^ Th17 cells are an osteoclastogenic population of CD4^+^ T cells^79^, ^80^, ^81^, ^82^ defined by their capacity to produce IL‐17.^83^ The presence of SFB in the intestine of healthy mice leads to lower bone density,^84^ which occurs via SFB‐driven expansion of Th17 cells in the gut, migration of Th17 cells to the bone marrow (BM) and increased secretion of IL‐17 in the BM.^85^, ^86^ These observations are a well‐characterized mechanism of how a gut microbe can act as a modulator of a host skeletal phenotype. These observations also offer a powerful model to establish proof of the principle that skeletal phenotypes are transmitted due to differential microbiome composition.

We further studied the effect of maternal GM on the skeletal maturation of offspring. We used CONV‐R and GF mice of two different stains C3H/HeN and C57BL/6 (BL6) purchased from TAC for this experiment. Both strains had differential microbiome compositions. BL6 mice were SFB^+^ while C3H/HeN (C3H) mice were SFB‐. GF mating pairs were generated for both strains of mice. Then, fecal material of each CONV‐R mouse strain was transferred to GF mating pairs of the other mouse strain, that is, fecal material from CONV‐R BL6 was transferred to GF C3H breeding pairs, and vice versa. We then assessed bone structure and turnover in 16‐week‐old female mice of the F1 generation produced by the colonized breeding pairs, which were colonized with the mother's microbiome from birth. We reported that at the age of 16 weeks old offspring of C3H mice colonized with the BL6 microbiome had lower femoral bone volume fraction BV/TV, Tb. Th, Tb. N and higher Tb. Sp, compared to the offspring of C3H mice having C3H microbiome. Interestingly, we did not see any bone anabolic effect in the offspring of BL6 mice colonized with the C3H microbiome.

**FIGURE 2:**
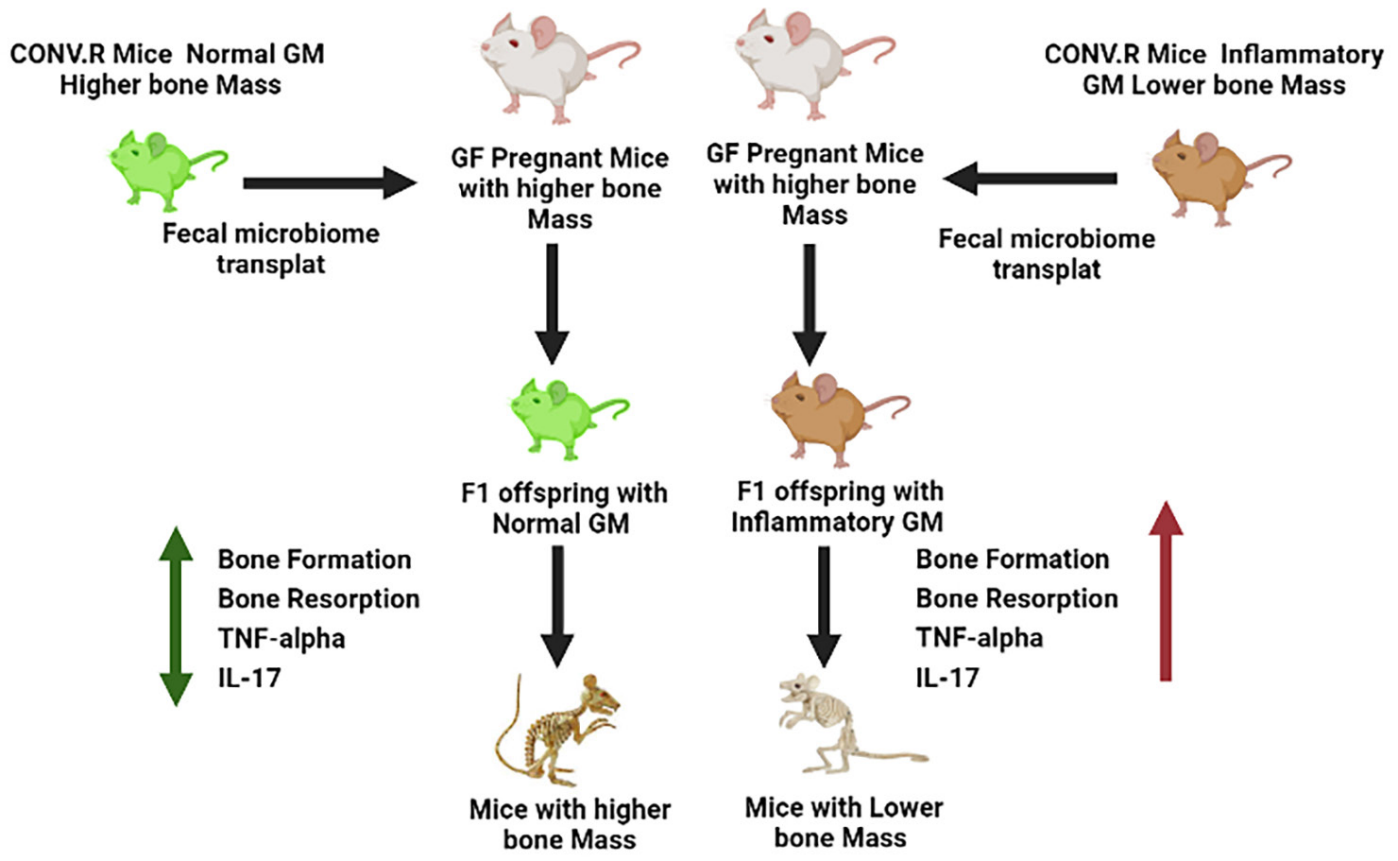
The effect of inflammatory gut microbiota on bone mass has been illustrated here. Inflammatory microbiome induces the differentiation of osteoclastogenic cytokines like RANKL, TNF‐α and IL‐17a. These cytokines induced osteoclast differentiation and bone resorption which leads to bone loss. Mice with inflammatory microbiome had lower bone mass and mice with healthy microbiome had higher bone mass with lower levels of circulatory inflammatory cytokines.

We further analysed the mRNA expression of inflammatory cytokines IL‐17 and TNF‐α in BM cells as well as the serum circulatory levels of both cytokines and we found that levels of these cytokines were higher in C3H mice offspring carrying BL6 microbiome. The serum levels of CTX were also higher in these mice indicating that the fecal material transfer effectively colonizes the BL6 microbiome in the GF mothers C3H mice. Furthermore, the colonized microbiome can be inherited in the offspring and affected the skeletal maturation of offspring by inducing bone resorption in these mice.^75^ Reports from us and other investigators strengthen the fact that GM is a critical non‐genomic factor that regulates bone mass in CONV‐R mice. The effect of inflammatory GM on bone mass has been illustrated in Figure 2.

## 5 GM REQUIRED TO INDUCE BONE LOSS IN SEX STEROID‐DEFICIENT MICE

Osteoporosis is the most common disease in developed countries,^87^ defined by the weakening of bones and increased fragility fracture due to increased bone resorption in sex steroid‐deficient conditions.^88^, ^89^, ^90^, ^91^, ^92^, ^93^ There were multiple mechanisms involved in inducing bone loss in sex steroid‐deficient conditions. For instance, increased osteoclast differentiation and life span,^94^ increased osteoblast apoptosis and decreased recruitment and life span,^95^ and increased reactive oxygen species (ROS)^96^, ^97^, ^98^ were a few well‐established mechanisms involved in sex steroid deficiency‐induced bone loss. Surprisingly, in the last three decades, a plethora of literature provided evidence that activated T cells play critical roles in the increased bone erosion in postmenopausal osteoporosis (PMO).^81^, ^82^, ^99^, ^100^, ^101^ However, scientists had a challenging question to address to completely imply the role of the immune system in sex steroid deficiency‐induced bone loss.

Sex steroid deficiency is a reproductive aging or metabolic pathological condition of bone loss and not an autoimmune condition. Therefore, the existence of activated T cells was a very big surprise for everyone and it was very imperative to find the antigen responsible for T cell activation in post‐menopausal osteoporotic conditions. For a long time, scientists failed to provide the source of the antigen responsible for T‐cell activation in PMO. In 2009, Braniste et al. reported that colonic permeability was increased in a rodent model of postmenopausal osteoporosis. These changes were associated with decreased expression of tight junction proteins occludin, members of the claudin family, and junction‐associated adhesion molecule (JAM) 3 in the colon.^102^ Furthermore, oestrogen receptor‐β (ER‐β) knockout mice exhibit decreased cell adhesion molecules and a disrupted tight junction in the colon as well as abnormal colon architecture.^103^ Additionally, colonic inflammation is associated with decreased ER‐β expression and increased colonic permeability.^104^ These studies suggested that oestrogen signaling is important in intestinal homeostasis. As GM exists in the intestinal lumen throughout and can be an important source of antigen required for T‐cell activation in sex steroid‐deficient conditions.

Besides, oestrogen is also a critical regulator of intestinal immune cell functions, loss of which has been shown to increase the expression of proinflammatory cytokines in immune cells isolated from the small intestine. Oestrogen receptors ER‐*α* and ER‐*β* are expressed to varying degrees in the gut‐associated immune cells.^105^, ^106^ Signalling through ER‐*α* in CD4^+^ T cells has been shown to have anti‐inflammatory properties inhibiting Th1/Th17 priming.^107^ Correspondingly, ER‐α‐deficient macrophages and dendritic cells express higher levels of TNFα in response to lipopolysaccharide.^108^ Together, these studies indicate that oestrogen deficiency could lead to local intestinal changes in intestinal permeability or intestinal barrier function that can lead to inflammatory cytokine expression akin to those seen in the blood and bone marrow. Therefore, these studies helped to morph the idea that sex steroid deficiency leads to increased gut permeability and provided the necessary antigen required for T‐cell activation in sex steroid deficiency.

Lie at al. showed that sex steroid deficiency in mice required GM to induce bone loss in mice. We showed that intestinal microbiota modulates inflammatory responses caused by sex steroid deficiency, leading to trabecular bone loss. In murine models, sex steroid deficiency increased gut permeability, expanded Th17 cells and upregulated the osteoclastogenic cytokines TNFα (TNF), RANKL and IL‐17 in the small intestine and the BM. However, in GF mice sex steroid deficiency failed to increase osteoclastogenic cytokine production, stimulate bone resorption and cause trabecular bone loss, demonstrating that the GM is central in sex steroid deficiency‐induced trabecular bone loss. Furthermore, we have shown that the serum levels of endotoxins of GM origin were higher in Lupron‐treated CONV‐R compared to control‐treated mice. The levels of endotoxins were minimum in GF mice.^109^ This work was very important as it answered a long‐standing question of antigens playing a critical role in the activation of T cells in PMO. However, there are more studies needed to demonstrate the role of GM‐produced endotoxins in T cell activation and bone loss in the OVX model or PMO patients. There is at least one study in the murine model that showed that treatment with berberine extracted from a Chinese plant protected OVX‐induced bone loss in rats. Berberine treatment was able to improve gut barrier function and serum levels of endotoxins in OVX rats. This positive effect was reflected by the lower number of pro‐inflammatory osteoclastogenic Th17 cells in BM and the circulation of OVX rats.^110^ However, these results still need to be confirmed by human studies.

## 6 MECHANISM OF ACTION BONE ANABOLIC ACTION OF LGG IN CONV‐R MICE

Probiotics are living bacteria that confer health benefits to the host when administered in an adequate amount. Probiotic bacteria are proposed to benefit human health mainly by three general mechanisms of action.^111^, ^112^ First, certain probiotics can exclude or inhibit pathogens, either through direct action or through influence on the commensal microbiota.^112^, ^113^ A second mechanism is the capacity of certain probiotic strains to enhance the epithelial barrier function by modulating signaling pathways, such as nuclear factor‐kB (NF‐kB), Akt and mitogen‐activated protein kinase (MAPK) ‐dependent pathways, which lead to for example the induction of mucus,^114^ or increased tight junction functioning.^115^ Third, most probiotic strains can also modulate host immune responses, exerting strain‐specific local and systemic effects.^116^ LGG, ATCC 53103 was originally isolated from fecal samples of a healthy human adult by Sherwood Gorbach and Barry Goldwin, explaining its typical surname letters GG. It was identified as a potential probiotic strain because of its resistance to acid and bile, good growth characteristics and adhesion capacity to the intestinal epithelial layer.^117^ Therefore, we tested the effect of treatment LGG on healthy mice. We treated 10 weeks old female BL6 mice with 1 × 10^9^ CFU for 4 weeks. We reported that LGG treatment resulted in a change in microbial diversity in the intestinal lumen and an expansion in the proportion of short‐chain fatty acids (SCFA) producing *clostridia species*. Furthermore, LGG supplementation of mice induced the enrichment of transcripts of a bacterial gene coding for *butyryl‐CoA: acetate CoA‐transferase*, an enzyme involved in butyrate production by lactate‐utilising bacteria in the gut. Consistent with this we found higher levels of butyrate in the gut as well as in serum in LGG‐treated mice as compared to vehicle‐treated mice.

**FIGURE 3:**
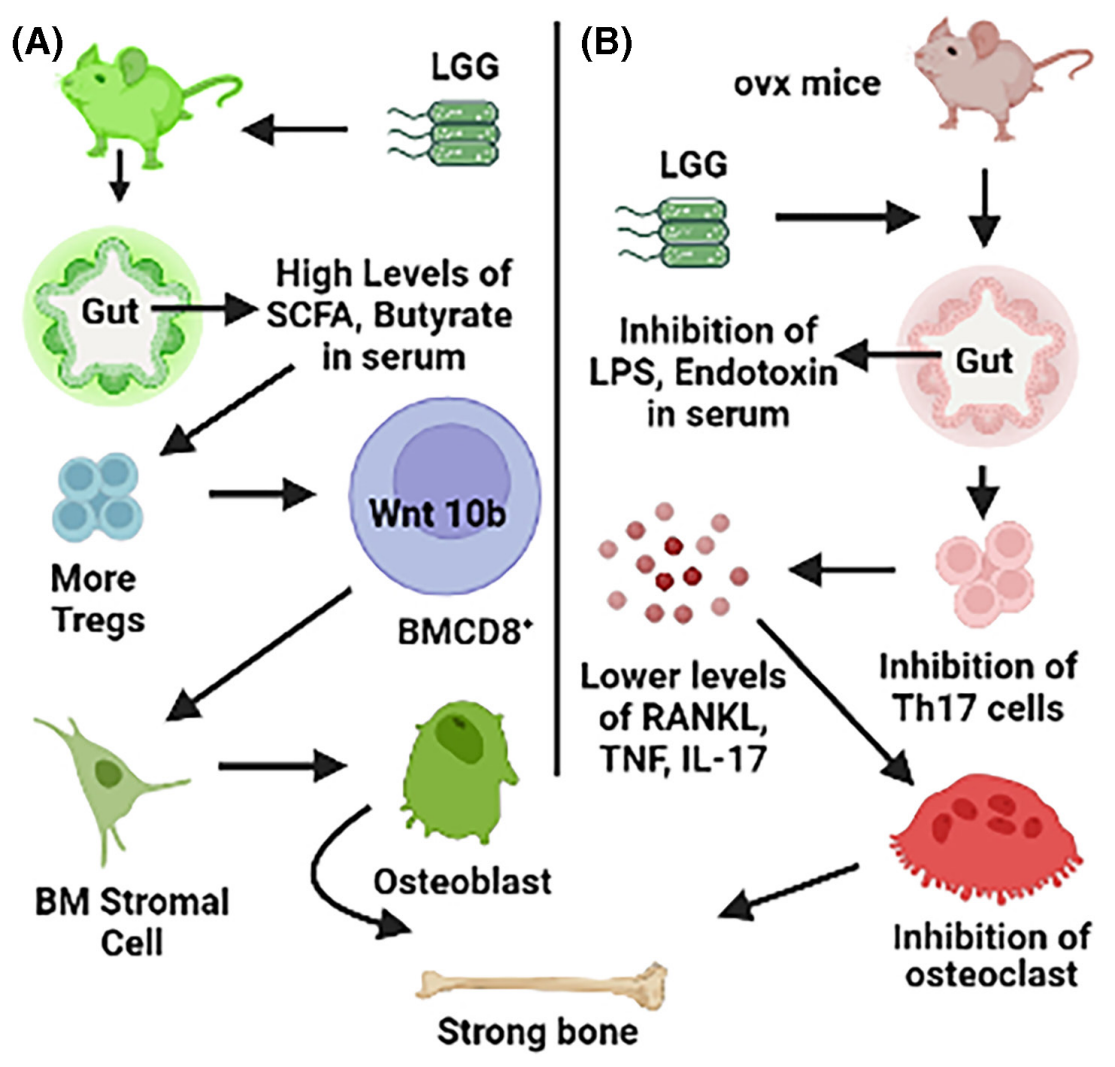
Mechanism of bone anabolic/protective effects of *Lactobacillus rhamnosus* GG (LGG) on bone mass in healthy (A) and ovariectomized (OVX) (B) mice. Healthy LGG‐fed mice had higher bone mass due to increased bone formation. The number of bone marrow (BM) Treg cells were higher in LGG‐fed mice compared to vehicle mice. Treg cells increased the production of Wnt 10b from BM CD8^+^ cells. CD8^+^ cells produced Wnt 10b‐induced Wnt signaling in BM stromal cells and increased their differentiation into bone forming osteoblast cells. In OVX mice, LGG ingestion inhibits the levels of lipopolysaccharide (LPS) and endotoxins of bacterial origin. Furthermore, LGG inhibits Th17 cells and TNF‐positive T cells in BM and inhibits osteoclast differentiation and function. This mechanism of anti‐inflammatory bone‐sparing effect of LGG in OVX mice is completely opposite to its effect in normal healthy mice.

Analysis of the trabecular region of the femur bone by μCT revealed higher bone volume fraction due to increased bone formation in LGG‐treated mice as compared to vehicle‐treated mice.^118^ Mechanistically, butyrate produced in the gut following LGG ingestion, or butyrate fed directly to GF mice, induced the expansion of intestinal and BM regulatory T (Treg) cells. Interaction of BM CD8^+^ T cells with Treg cells resulted in increased secretion of Wnt10b, a bone anabolic Wnt ligand. Treg cells promoted the assembly of an NFAT1‐SMAD3 transcription complex in CD8^+^ cells, which drove the expression of *Wnt10b* mRNA from CD8^+^ cells. Wnt10b secreted from BM CD8^+^ cells acts on BM stromal cells and activates Wnt signaling in BM stromal cells. Activation of Wnt signaling in BM stromal cells induces their differentiation in osteoblast and hence induces bone formation in LGG‐treated mice.^118^ The Mechanism of anabolic effect of LGG on bone has been illustrated in Figure 3.

## 7 MECHANISM OF ANTI‐RESORPTIVE EFFECT OF LGG IN OVX MICE

Postmenopausal osteoporosis is the leading cause of fracture and morbidity in older individuals. OVX in mice is the gold standard model to study the effect of sex steroid deficiency‐induced bone loss. Therefore, we investigated the effect of LGG ingestion on bone mass in 10 weeks old OVX mice. The Mechanism of antiresorptive effect of LGG on bone has been illustrated in Figure 3. Treatment with LGG for 4 weeks protected OVX‐induced bone loss in mice as compared to vehicle‐treated mice. Mechanistically, LGG treatment in OVX mice restored intestinal barrier function by reinstating the gut permeability and inhibiting the levels of proinflammatory osteoclastogenic cytokines TNF‐α and IL‐17.^109^ Another group also reported the effect of *Lactobacillus rhamnosus* (LR) species on bone mass in OVX BALB/C mice. They reported LR inhibits osteoclastogenesis and modulates the differentiation of Treg‐Th17 cells under in vitro conditions. We further observed that LR attenuates bone loss in OVX mice. Both the cortical and trabecular bone mass of LR‐treated OVX mice were significantly higher than vehicle‐treated OVX mice. Mechanistically, the percentage of osteoclastogenic Th17 cells at distinct immunological sites such as BM, spleen, LN and PP was significantly reduced, whereas the percentage of anti‐osteoclastogenic Tregs were significantly enhanced in LR‐treated mice thereby preventing bone loss.^119^

## 8 CONCLUSION, FUTURE AVENUES OF EXPLOITING GM AND PROBIOTICS FOR HEALTH BENEFITS

Alteration in the GM in MS and related chronic health defects is among the foremost errands of microbiome research and needs for clinical use of probiotics. In the last decade, evidence lacks for the implications for microbiome modification to improve metabolic health in particular when applied impersonalized. However, very recently several studies showed that personalized bacterial strain can be used for health benefits and probiotics have tremendous potential in personalized nutrition and medicine to develop healthy diets.^118^, ^120^ Personalized or precision medicine is an innovative clinical tactic battered to the individual patient and founded on the incorporation of clinical, genetic, and environmental factors that define a patient uniquely from other individuals featuring similar clinical symptoms. We believe that microbiome is very important in providing the individual bacterial, fungal or viral strain to treat different pathological condition. Precise microbiome can be used in medicine to treat not only intestine‐related disorders but can also be used to improve the health of skin, liver, brain and other distant organ systems. There are several reports, supports the notion of using probiotics in precise medicine such as some strains of *Lactobacillus* used to prevent hyperglycaemia, alveolar bone loss and inflammation in murine models of inflammation, diabetes and periodontitis^121^, ^122^ and osteoporosis.

Fractures due to osteoporosis have devastating consequences, particularly in the elderly. Vertebral fractures are a source of significant pain and crippling, while complications of hip fracture and the major surgery necessitated, lead to mortality rates of 24%–30% in the first year alone. Furthermore, almost 50% of survivors suffer permanent disability.^123^, ^124^, ^125^, ^126^ Fracture rates increase rapidly with age culminating in a lifetime risk of fracture of about 40% in 50‐year‐old women, a rate similar to that of coronary heart disease. The prevalence of osteoporosis in the United States alone was estimated at 10 million in 2005 with an estimated 2 million fractures at an economic cost of more than $19 billion. Annual fractures and costs in the US are projected to rise by almost 50% by 2025.^124^

Gut microbiota is a major environmental factor that regulates bone mass in humans and animals. Knowledge of GM modulation can be used to achieve peak bone mass in early life so that one can have higher bone mass at the beginning of reproductive aging in females and males. Supplementation of probiotics can protect against sex steroid deficiency‐induced bone loss in human osteoporotic patients. Unfortunately, there is a need for clinical trials to check the efficacy of all positive effects of GM in PMO patients. There can be some other uses of inflammatory GM such as one can use of highly inflammatory probiotic bacteria to induce inflammation and activation of the immune system in several carcinomas. Therefore, there is a huge potential for GM to be used for greater health benefits in the future.

## AUTHOR CONTRIBUTIONS

AMT drafted all sections of the manuscript, write the manuscript and made the figures. AMT drafted sections of the manuscript.

## FUNDING INFORMATION

The authors were funded by CSIR‐CDRI, an internal institute grant.

## CONFLICT OF INTEREST STATEMENT

Author has no conflict of interest to declare.

## Data Availability

None declared.
